# GATA binding protein 3 is correlated with leptin regulation of PPARγ1 in hepatic stellate cells

**DOI:** 10.1111/jcmm.13002

**Published:** 2016-10-06

**Authors:** Wei Guan, Fangyun Cheng, Hao Wu, Qing Cao, Xiaofei Zhu, Yan Fan, Huixia Zhu, Yajun Zhou

**Affiliations:** ^1^ Department of PharmacologySchool of PharmacyNantong UniversityNantongJiangsuChina; ^2^ Department of Biochemistry & Molecular BiologyMedical CollegeNantong UniversityNantongJiangsuChina

**Keywords:** leptin, GATA binding protein 3, peroxisome‐proliferator activated receptor γ1, liver fibrosis, hepatic stellate cell

## Abstract

Accumulating evidence reveals that hormone leptin, mainly produced by adipocyte, plays a unique role in promotion of liver fibrosis. Hepatic stellate cell (HSC) activation is a key step in liver fibrosis and peroxisome‐proliferator activated receptor γ (PPARγ) exerts a crucial role in inhibition of HSC activation. Our previous researches demonstrated that leptin reduced PPARγ1 (a major subtype of PPARγ in HSCs) expression through GATA binding protein 2 (GATA2) binding to a site around −2323 in PPARγ1 promoter. The present researches aimed to examine the effect of GATA3 on leptin‐induced inhibition of PPARγ1 and elucidate the relationship between GATA3 and GATA2. Gene expressions were analysed by real‐time PCR, western blot, luciferase assay and immunostaining. C57BL/6J ob/ob mouse model of thioacetamide‐induced liver injury was used *in vivo*. Results demonstrate that leptin significantly induces GATA3 expression in HSCs by multiple signalling pathways including NADPH oxidase pathway. There exist crosstalks between NADPH oxidase pathway and the other pathways. GATA3 can bind to GATA2‐binding site in PPARγ1 promoter and interacts with GATA2, contributing to leptin inhibition of PPARγ1 expression in HSCs. These data demonstrated novel molecular events for leptin inhibition of PPARγ1 expression in HSCs and thus might have potential implications for clarifying the detailed mechanisms underlying liver fibrosis in diseases in which circulating leptin levels are elevated such as non‐alcoholic steatohepatitis in obese patients.

## Introduction

Since liver fibrosis is shown to be six times more prevalent in obese patients as compared with general population 1, 2 and obesity is often accompanied by hyperleptinaemia 3, 4, the effects of leptin, an adipocyte‐derived hormone, on liver fibrogenesis are investigated and evidences indicate a unique role of leptin in promoting liver fibrogenesis *in vitro* and *in vivo* 5, 6, 7, 8, 9. Activated hepatic stellate cells (HSCs) are the main cells for the production of extracellular matrix (ECM) and HSC activation is a key step in liver fibrogenesis 10. Peroxisome‐proliferator activated receptor γ (PPARγ), a transcription factor (TF) controlling adipocyte differentiation, was demonstrated to function as a crucial TF in inhibiting HSC activation 11, 12 and ectopic expression of PPARγ can cause the morphological and biochemical reversal of activated HSCs to quiescent cells 13.

Based on those results, we investigated the mechanisms underlying the role of leptin in liver fibrogenesis and are focused on the effect of leptin on PPARγ in HSCs. Our results showed that leptin response region existed between position −2333 and −2245 upstream of the transcription start site of mouse PPARγ1 promoter and leptin exerted an inhibitory effect on the expression of PPARγ1, a major subtype of PPARγ in HSCs 14, thus contributing to HSC activation 15. Furthermore, we revealed that GATA binding protein‐2 (GATA2) could bind to a site around position −2323 in leptin response region in mouse PPARγ1 promoter and mediated leptin inhibition of PPARγ1 in HSCs 15.

GATA3 was demonstrated to enhance the development of pulmonary fibrosis 16. Moreover, Tong and colleague demonstrated that GATA3 seemed able to bind to GATA2‐binding site 17. Though the sequence of GATA2‐binding site in PPARγ1 promoter shown by us 15 is different from that shown by Tong 17, there is a possibility that GATA3 binds to GATA2‐binding site on PPARγ1 promoter and thereby affects PPARγ1 expression. Thus, the purpose of the present study is to examine whether GATA3 is correlated with the effect of leptin on PPARγ1 in HSCs and explore whether GATA3 binds to the GATA2‐binding site around position −2323 in mouse PPARγ1 promoter. And if so, the relationship between GATA2 and GATA3 will be elucidated.

## Materials and methods

### Materials

Leptin was purchased from ProSpec‐Tany TechnoGene (Rehovot, Israel). XAV939 (a specific inhibitor for β‐catenin signalling pathway) was purchased from Santa Cruz (Santa Cruz, CA, USA). Diphenyleneiodonium chloride (DPI, an inhibitor for NADPH oxidase signalling pathway) and thioacetamide (TAA) were from Sigma‐Aldrich (St. Louis, MO, USA). Ly294002 (a specific inhibitor for phosphoinositide 3‐Kinase, PI3K) and cyclopamine (a specific inhibitor for Hedgehog signalling pathway) were from selleck Chemicals (Houston, TX, USA).

### HSC isolation and culture

Hepatic stellate cells were isolated from adult Kunming mice (Animal Research Center of Nantong University, Nantong, China) as we described previously 18. Hepatic stellate cells between passages 3 and 6 were used for experiments. Hepatic stellate cells underwent 12 hrs of serum starvation in DMEM with 1% foetal bovine serum before stimulation with 100 ng/ml of leptin 9.

### Animal treatment

Thioacetamide is usually used for induction of mouse liver injury 9, 19 and thereby a mouse model of TAA‐induced liver damage was adopted 19. C57BL/6J ob/ob mice (leptin‐deficient obese mouse, 6 weeks old, Model Animal Research Center of Nanjing University, Nanjing, China) were randomly separated into two groups (six mice/each group). The first two groups were treated with leptin (1 μg/g body weight, once per day) or vehicle throughout the 4‐week period of TAA (200 μg/g body weight, two times a week) by intraperitoneal injection (i.p.) 9, 19. The second two groups were received DPI (1 μg/g body weight, once per day) 20 or vehicle by i.p. throughout the 4‐week period of treatment with TAA plus leptin. After 4‐week, the livers were fixed in 4% buffered paraformaldehyde for immunofluorescence staining or sirius red staining. The experiments were approved by the Ethical Committee of the University of Nantong (2012‐0031).

### Immunofluorescence and sirius red staining

Double fluorescence staining was performed with liver sections as we described previously 9. Briefly, paraformaldehyde‐fixed liver sections were blocked with normal serum and incubated with primary antibody against 4‐hydroxynonenal (4‐HNE, 1:50; Abcam, Cambrige, MA, USA), Sonic hedgehog (Shh, 1:50; Santa Cruz), Ser 473 phosphorylated AKT (p‐AKT, 1:100; Cell Signaling Technology Inc, Bevely, MA, USA), β‐catenin (1:100; Santa Cruz), PPARγ (1:100, Abcam), GATA3 (1:50; Santa Cruz) and primary antibody against synaptophysin (SYP, 1:10; Abcam), a marker for quiescent and activated HSCs 21, followed by incubation with DyLight594‐conjugated secondary antibody (1:500; ImmunoReagents, Inc, Raleigh, NC, USA) and DyLight488‐conjugated secondary antibody (1:500; ImmunoReagents, Inc). The images were captured with the fluorescence microscope and representative images were shown.

Sirius red was used to stain collagen on liver. Briefly, mouse liver sections were stained with picric acid‐fast green (Amresco, Solon, OH, USA) and then incubated with picric acid–sirius red (Amresco) for 1 hr. The images were captured with light microscope and representative images were shown (Data S2).

### Plasmid constructs and transient transfection assay

Luciferase reporter plasmid pPPARγ1(−2333) Luc contained mouse PPARγ1 promoter region between −2333 and +157) as we previous described 15. Reporter plasmid pPPARγ1(GATA mut)Luc contained GATA2 binding site‐mutated PPARγ1 promoter (around the site of −2323) 15. The site was mutated by changing the sequence GATA into TTTA 15. Reporter plasmid p3×GATALuc contained three tandem repeats of potential GATA2‐binding site in PPARγ1 promoter and tandem repeats was 3× (5′‐tcttttGATAtgtgcaga‐3′) 15.

To construct mouse GATA3 promoter luciferase reporter plasmid pGATA3(−2657)Luc, GATA3 promoter (from −2531 to +194) was amplified from genomic DNA of Kunming mice and was inserted into MluI/XhoI sites of pGL3‐basic. The primers used for construction of pGATA3(−2657)Luc were shown in Data S1 and the sequences of the were confirmed by DNA sequence analysis.

Hepatic stellate cells in twelve‐well plastic plates (unless otherwise stated) were transiently transfected with the respective plasmid by using LipofectAMINE reagent (Life Technologies, New York, NY, USA) according to manufacturer's instructions. Reporter plasmids were cotransfected into the cells with 30 ng of control vector expressing Renilla luciferase (pRL‐TK; Promega, Madison, WI, USA). Luciferase activity was quantified fluorimetrically by using the Dual‐Luciferase Reporter Assay System (Promega) and the data were expressed as the ratios of Photinus to Renilla luciferase activity.

### Real‐time PCR

Total RNA was extracted by TRI‐Reagent (Sigma‐Aldrich) according to the manufacturer's instructions and real‐time PCR was performed as we described previously 22. For analysis of fold change in target gene mRNA level relative to the endogenous cyclophilin control, the Ct values were normalized against cyclophilin and analysed by using the ΔΔCt method. All the primers were shown in Data S1.

### Western blot analysis and immunoprecipitation assay

Western blot analyses were performed as described previously 18. Briefly, target protein was detected by primary antibody against GATA3 (1:500; Santa Cruz), PPARγ (1:500; Abcam), α1(I)collagen (1:2000; Santa Cruz), α‐smooth muscle actin (α‐SMA, 1:2000; Abcam), Shh (1:500; Santa Cruz), or β‐actin (1:2000; Santa Cruz) and by horseradish peroxidase‐conjugated secondary antibody (1:4000). The target protein bands were densitometrically determined by Quantity One 4.4.1 (Bio‐Rad, Hercules, CA, USA) and the numbers beneath the blots indicated the fold changes in the band densities relative to the control (the first band on the left) after normalization with the internal control β‐actin.

For immunoprecipitation assay, 293T cells cotransfected with pcDNAGATA2 (encoding mouse GATA2 protein) 15 and pFlag‐GATA3 (encoding Flag‐GATA3 protein; Addgene, Cambrige, MA, USA) were washed by phosphate‐buffered saline and were lysed by RIPA buffer. Some of the cell lysate was used as input sample and the other were precleared with protein A/G Agarose (Santa Cruz). The cleared cell lysate was incubated with the anti‐Flag antibody (Abbkine, Inc, Redlands, CA, USA) (or the control normal IgG; Santa Cruz) for 1 hr at 4°C and then with protein A/G Agarose for 12 hrs at 4°C. Precipitated immune complexes were detected by western blot analysis by using primary antibody against GATA2 (1:500; Santa Cruz) or GATA3 (1:500; Santa Cruz) and by horseradish peroxidase‐conjugated secondary antibody.

### Electrophoretic mobility shift assay

Electrophoretic mobility shift assay (EMSA) assays were conducted by LightShift Chemiluminescent EMSA Kit (Pierce Biotechnology, Rockford, IL, USA) as described previously 15. Briefly, Biotinylated DNA fragments (Data S1, containing the GATA2‐binding site around −2323 in mouse PPARγ1 promoter) was synthesized by Life Technologies (Shanghai, China) for GATA3 binding assay. 5 μg protein of nuclear extract from cells treated with leptin was incubated with the labelled probes. For the competition assay, 5 μg protein of nuclear extract was incubated with 100‐fold molar excess of the unlabelled probes before addition of the labelled probe. For supershift assay, 5 μg protein of nuclear extract was incubated with 1 μg of GATA3 antibody before addition of the labelled probe. The samples were subjected to electrophoresis in a 5% nondenatureating polyacrylamide gel and transferred onto a nylon membrane. The samples were detected by Substrate Working Solution.

### Chromatin immunoprecipitation

Chromatin immunoprecipitation (ChIP) assays were carried out as we described previously 23 by using Pierce Agarose Chip Kit (Pierce Biotechnology). Briefly, the nuclei from the cross‐linked cells with 1% formaldehyde were incubated with Nuclease. Some of the digested chromatin was preserved as input control and the rest of the digested chromatin were incubated with GATA3 antibody (Santa Cruz) or normal IgG (as a control). The purified DNA from immunoprecipitation and the input samples were used to amplify a fragment (132 bp) between −2362 and −2230 (containing the GATA2‐binding site) by PCR. The used primers were shown in Data S1. The PCR products were examined by 2% agarose gel electrophoresis.

### Statistical analysis

The results are expressed as mean values ± S.D. Comparisons of multiple treatment conditions with controls were analysed by anova with the Dunnett's test for *post hoc* analysis. Differences between means were evaluated using an unpaired two‐sided Student's *t*‐test. Each result was obtained from at least three independent differentiation experiments. A *P*‐value of less than 0.05 is considered as significant.

## Results

### Leptin promotes GATA3 expression

**Figure 1:**
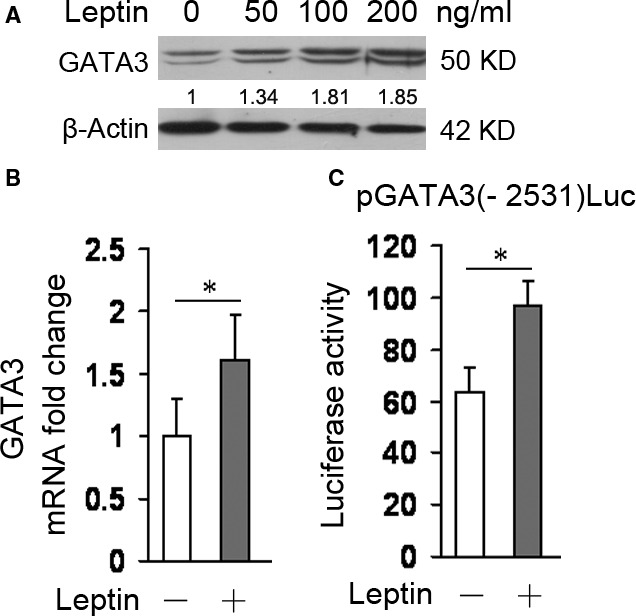
Leptin induces GATA3 expression. (**A** and **B**) Detection of GATA3 expression at protein and mRNA levels (*n* = 3). Serum‐starved HSCs were incubated with or without increased doses of leptin (**A**) or 100 ng/ml of leptin (or the vehicle, −) (**B**) for 24 hrs and GATA3 protein and mRNA levels were examined by western blot analyses and real‐time PCR, respectively. *P < 0.05. (**C**) Examination of GATA3 promoter activity by luciferase assay (*n* = 3). HSCs were transfected with 1.6 μg of GATA3 promoter reporter plasmid pGATA3(−2531)Luc and then underwent 12 hrs of serum starvation before addition of 100 ng/ml of leptin (or the vehicle, −) for another 24 hrs. Luciferase assay was performed. *P < 0.05.

Firstly, we examined the effect of leptin on GATA3 expression in HSCs. Hepatic stellate cells were incubated with leptin and the levels of GATA3 protein and mRNA were evaluated by western blot and real‐time PCR, respectively. As shown in Figure 1A and B, leptin treatment clearly increased the levels of GATA3 protein and mRNA. We also transfected HSCs with GATA3 promoter reporter plasmid and luciferase assay indicated that leptin stimulated GATA3 promoter activity (Fig. 1C).

These results demonstrated that leptin could up‐regulate GATA3 expression and increase GATA3 promoter activity in HSCs *in vitro*.

### GATA3 down‐regulates PPARγ1 expression and up‐regulates the expressions of α‐SMA and α1(I) collagen

**Figure 2:**
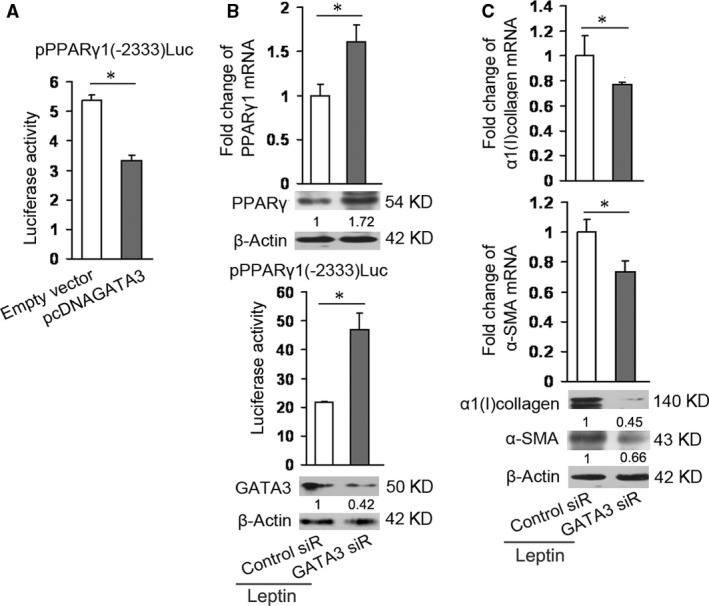
GATA3 reduces PPARγ1 expression and increases α‐SMA and α1(I) collagen expressions. (**A**) Examination of PPARγ1 promoter activity by luciferase assay (*n* = 3). HSCs were cotransfected with 0.8 μg/ml of pcDNAGATA3 (or empty vector) plus 0.8 μg/ml of pPPARγ1(−2333)Luc and then incubated for 24 hrs. Luciferase assay was performed. *P < 0.05. (**B**) Detection of PPARγ1 expression by western blot analysis, real‐time PCR, and luciferase assay, respectively (*n* = 3). The first group of HSCs in 6‐well plate were transfected with 1 μg of GATA3 siRNA (GATA3 siR) or the control siRNA (Control siR) and then incubated for 48 hrs in the presence of 100 ng/ml of leptin for assessing PPARγ1 mRNA levels and protein levels by western blot and real‐time PCR analyses, respectively. The second group of HSCs in 12‐well plate were transfected with 1 μg of pPPARγ1(−2333)Luc plus 0.6 μg of GATA3 siRNA (GATA3 siR) or the control siRNA (Control siR) and then incubated for 48 hrs in the presence of 100 ng/ml of leptin for luciferase assay. Western blot analysis in the lower panel demonstrated GATA3 expression. *P < 0.05. (**C**) Detection of the expressions of α‐SMA and α1(I) collagen by real‐time PCR and western blot analyses, respectively (*n* = 3). HSCs in 6‐well plate were cotransfected with 1 μg of GATA3 siRNA (GATA3 siR) or the control siRNA (Control siR) and then incubated for 48 hrs in the presence of 100 ng/ml of leptin for assessing mRNA and protein levels of α‐SMA and α1(I) collagen by real‐time PCR and western blot analyses, respectively. *P < 0.05.

For detecting whether GATA3 affected PPARγ1 expression, HSCs were cotransfected with PPARγ1 promoter reporter plasmid pPPARγ1(−2333)Luc (containing wild‐type PPARγ1 promoter) 15 and mouse GATA3 expression plasmid pcDNAGATA3 (a gift Dr. Morisada Hayakawa, Jichi Medical University, Japan). Luciferase assay showed a decrease in PPARγ1 promoter activity in HSCs transfected with pcDNAGATA3 (Fig. 2A). We also used GATA3 siRNA (Santa Cruz) to reduce GATA3 protein levels in HSCs and then analysed PPARγ1 promoter activity, PPARγ1 mRNA level, and PPARγ protein level. Figure 2B showed that knockdown of GATA3 led to significant increases in PPARγ1 promoter activity, PPARγ1 mRNA level, and PPARγ protein level. The results shown in Figure 2A and B indicated that GATA3 inhibited PPARγ1 expression in HSCs. Since PPARγ exerts an important role in inhibiting HSC activation, the effects of GATA3 on the expressions of α‐SMA (a well‐established marker for HSC activation) and α1(I) collagen (the major component of ECM) in HSCs were evaluated. Knockdown of GATA3 expression reduced the expressions of α‐SMA and α1(I) collagen at mRNA level and protein level (Fig. 2C).

### GATA3 binds to GATA2‐binding site on PPARγ1 promoter and interacts with GATA2

Base on leptin‐induced promotion role in GATA3 expression and the inhibitory effect of GATA3 on PPARγ1 promoter, we want to know whether GATA3 also bound to GATA2‐binding site in leptin response region in PPARγ1 promoter. Thus, we performed EMSA by using the GATA2‐binding site in PPARγ1 promoter as a probe and by using nuclear extract from HSCs stimulated by leptin. Figure 1A indicated that 1 μg of antibody against GATA3 markedly reduced the shift band formation. As expected, 1 μg antibody against GATA2 affected the shift band formation and the same effect was demonstrated by using 0.5 μg of GATA3 antibody plus 0.5 μg of GATA2 antibody. These results suggested that GATA3 could bind to GATA2‐binding site around −2323 in PPARγ1 promoter.

**Figure 3:**
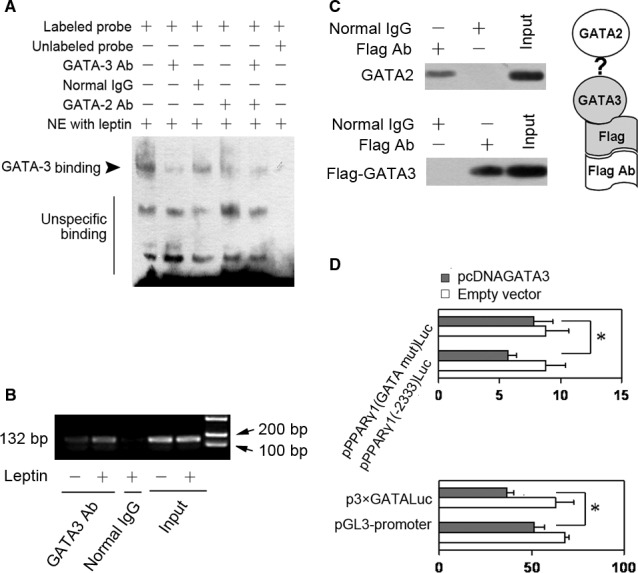
GATA3 binds to GATA2‐binding site on PPARγ1 promoter and interacts with GATA2. (**A**) EMSA of GATA3 binding to GATA2‐binding site on PPARγ1 promoter (*n* = 3). After HSCs were stimulated with 100 ng/ml of leptin for 24 hrs, nuclear extracts (NE) were prepared and 5 μg of nuclear proteins were incubated with biotinylated DNA fragment (containing the GATA‐2‐binding site around −2323). For competition assay, 5 μg of nuclear proteins were preincubated with 100‐fold molar excess of the unlabelled probe before addition of labelled probe. For supershift assay, 5 μg of nuclear proteins were preincubated with 1 μg of anti‐GATA3 antibody, or 1 μg of anti‐GATA2 antibody, or 1 μg of normal IgG, or 0.5 μg of anti‐GATA3 antibody plus 0.5 μg of anti‐GATA2 antibody before addition of the labelled probe. A representative EMSA was shown. (**B**) ChIP analysis of GATA3 binding to the site (around −2323) of PPARγ1 promoter (*n* = 3). HSCs were stimulated with leptin or the vehicle (−) for 24 hrs and ChIP analysis was performed by using anti‐GATA3 antibody or normal IgG as described in materials and methods. The purified DNA from immunoprecipitation and from the input samples were used to amplify a fragment (132 bp) between −2362 and −2230 (containing the GATA‐2‐binding site) by PCR and the PCR products were examined by 2% agarose gel electrophoresis. (**C**) Immunoprecipitation assay of the interaction between GATA3 and GATA2 (*n* = 3). After pFlag‐GATA3 (encoding Flag‐GATA3 protein) were cotransfected 293T cells with pcDNAGATA2 (encoding mouse GATA2 protein) and incubated for 24 hrs, 293T cells were lysed by RIPA buffer and the some of the cell lysate was used as input sample and the others were used for immunoprecipitation by using the anti‐Flag antibody or the normal IgG. Precipitated immune complexes were detected by western blot analysis by using antibody against GATA2 or GATA3. A representative result was shown. (**D**) Luciferase assay (*n* = 3). The first group of HSCs were cotransfected with 0.8 μg of pPPARγ1(GATA mut)Luc or 0.8 μg of pPPARγ1(−2333)Luc plus 0.8 μg of pcDNAGATA3 or 0.8 μg of the empty vector and then incubated for 24 hrs. The second group of HSCs were cotransfected with 0.8 μg of p3×GATALuc or pGL3‐promoter vector (control) plus 0.8 μg of pcDNAGATA3 or empty vector and then incubated for 24 hrs. Luciferase assay was performed. *P < 0.05.

Chromatin immunoprecipitation assay was used to validate the results from EMSA. The purified DNA from immunoprecipitation with GATA3 antibody was used to amplify a fragment (132 bp) between −2362 and −2230 (containing the GATA2‐binding site) by PCR. The PCR products were examined by agarose gel electrophoresis. Figure 3B showed that GATA3 antibody led to clear band, suggesting that leptin promoted GATA3 binding to GATA2‐binding site in PPARγ1 promoter *in vivo*. Thus, the results led us to investigate whether GATA3 interacted with GATA2 by immunoprecipitation assay. GATA2 and Flag‐GATA3 were overexpressed in 293T cells and the cell lysate was incubated with the anti‐Flag antibody. Precipitated immune complexes were detected by western blot analysis by using primary antibody against GATA2 or GATA3. As shown in Figure 3C, GATA2 and GATA3 were clearly detected by western blot, suggesting that there existed an interaction between GATA3 and GATA2.

Furthermore, reporter plasmid pPPARγ1(GATA mut)Luc or the reporter plasmid pPPARγ1(−2333)Luc was used to cotransfected HSCs with pcDNAGATA3 or the empty vector. Luciferase assay (the upper panel in Fig. 3D) demonstrated that the mutation of GATA2‐binding site reduced the inhibitory effect of GATA3 on PPARγ1 promoter. Next, p3×GATALuc or pGL3‐promoter vector (control) was used to cotransfected HSCs with pcDNAGATA3 or the empty vector. Luciferase assay (the lower panel in Fig. 3D) showed that GATA3 reduced the luciferase activity in HSCs with p3×GATALuc as compared with the sample with empty vector. These results were in line with those shown in EMSA and in ChIP assay.

### Multiple signalling pathways mediates leptin regulation of GATA3 expression

**Figure 4:**
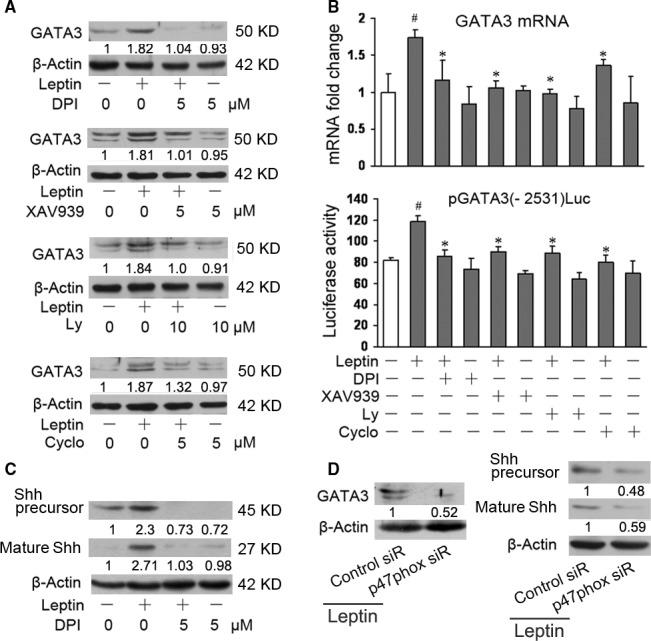
Multiple signalling pathways mediate leptin regulation of GATA3 expression. (**A**) Western blot analysis of GATA3 expression (*n* = 3). Serum‐starved HSCs were preincubated with the respective inhibitor (Diphenyleneiodonium, DPI; XAV939; Ly294002, Ly; Cyclopamine, Cyclo) or the vehicle (0) for 1 hr before addition of 100 ng/ml of leptin or the vehicle (−) for another 24 hrs. Western blot analysis was performed. (**B**) Real‐time PCR of GATA3 expression and luciferase assay of GATA3 promoter activity. The first group of HSCs were treated with the respective inhibitor and leptin (or the vehicle) as in (**A**) and real‐time PCR was performed (the up panel). The second group of HSCs were transfected with 1.6 μg of pGATA3(−2531)Luc and then treated with the respective inhibitor and leptin (or the vehicle) as in (**A**). Luciferase assay was performed (the lower panel). #*P* < 0.05 *versus* the cells without treatment (the first column on the left). *P < 0.05 *versus* the cells with leptin alone (the second column on the left). (**C** and **D**) Western blot analysis of Shh and GATA3 expressions (*n* = 3). The first group of serum‐starved HSCs were preincubated with the DPI (or the vehicle, 0) for 1 hr before addition of 100 ng/ml of leptin (or the vehicle, −) for another 24 hrs. Western blot analysis was performed (**C**). The second group of HSCs in six‐well plate were transfected with 1 μg of p47phox siRNA (p47phox siR) or the control siRNA (control siR) and incubated for 48 hrs in the presence of 100 ng/ml of leptin. Western blot analysis was performed (**D**).

Next, we detected the signalling pathways which were involved in leptin regulation of GATA3 expression in HSCs. The signalling pathways of PI3K/AKT pathway, β‐catenin, and Shh (one of three types of protein hedgehog which regulate cell fate decision) can mediate leptin‐induced inhibition of PPARγ in HSCs 15, 22 whereas NADPH oxidase pathway appears to play the key role in activating many signalling pathways 24, 25 and can be induced by leptin in HSCs 26, thus the researches were focused on these signalling pathways. The cells were incubated with different inhibitor in the presence or absence of leptin. Western blot analyses (Fig. 4A) and real‐time PCR (the up panel in Fig. 4B) showed that the inhibitor for NADPH oxidase pathway, β‐catenin pathway, PI3K/AKT pathway, or Shh pathway reduced leptin promotion GATA3 expression, suggesting that these signalling pathways were correlated with the effect of leptin on GATA3. Furthermore, HSCs were transfected with GATA3 promoter reporter plasmid pGATA3(−2657)Luc and incubated with the respective inhibitor in the presence or absence of leptin. Luciferase assay indicated that inhibition of the respective signalling pathway led to the decrease in leptin‐induced GATA3 promoter activity (the lower panel in Fig. 4B).

p47phox, a subunit of NADPH oxidase, plays a central role in the activity of NADPH oxidase 27, thus, we also transfected HSCs with p47phox siRNA (Santa Cruz) and western blot analysis demonstrated the same effect of NADPH oxidase pathway on GATA3 (the left panel in Fig. 4D).

In view of the key role of NADPH oxidase signal in activating many signalling pathways 24, 25, we examined the effects of NADPH oxidase signalling on the other signalling. Figure 4C revealed that inhibition of leptin‐induced NADPH oxidase pathway significantly reduced the protein levels of Shh precursor and mature Shh, suggesting a crosstalk between NADPH oxidase pathway and Shh pathway. Knockdown of p47phox demonstrated the same results as shown in Figure 4C (the right panel in Fig. 4D).

Our recent researches showed that inhibition of NADPH oxidase pathway by DPI or knockdown of p47phox by p47phox siRNA reduced leptin‐induced β‐catenin pathway in HSCs *in vitro* (unpublished data). Minicis showed that NADPH oxidase pathway mediates leptin‐induced PI3K/AKT pathway in HSCs *in vitro* 26.

### Leptin increases the levels of 4‐HNE, p‐AKT and GATA3 in HSCs in ob/ob mouse model of TAA‐induced liver injury

**Figure 5:**
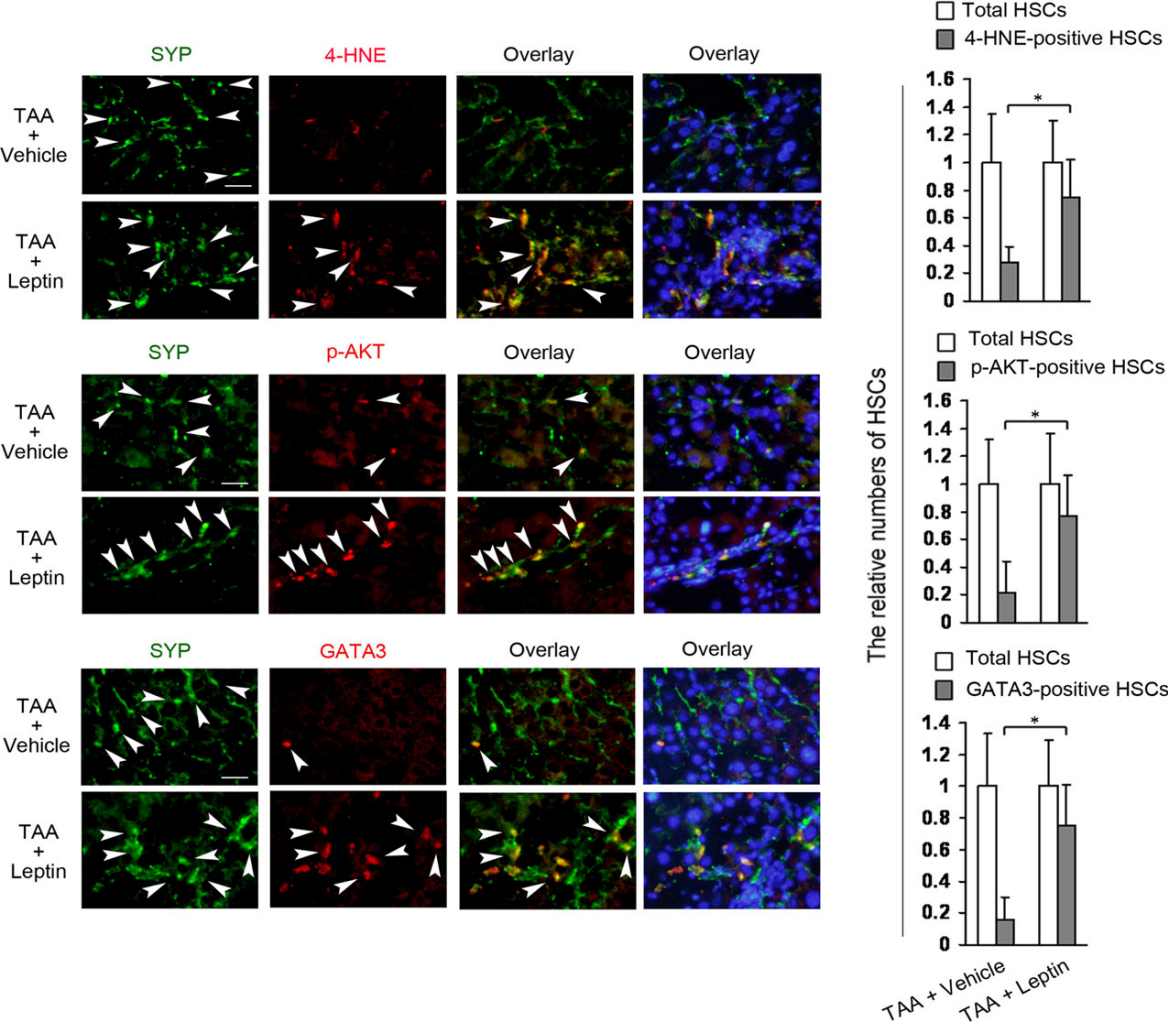
Leptin increases the levels of 4‐HNE, p‐AKT, and GATA3 in HSCs in ob/ob mouse model of TAA‐induced liver injury. Two groups of ob/ob mice (6 mice/each group) were received TAA (200 μg/g body weight, two times a week) plus vehicle (TAA+Vehicle) or TAA plus leptin (1 μg/g body weight, once per day) (TAA+Leptin) by intraperitoneal injection (i.p.) for 4‐week. Double fluorescence staining on the section of liver was performed for detecting 4‐HNE‐, p‐AKT‐, or GATA3‐positive HSCs by using the respective primary antibody plus primary antibody against synaptophysin (SYP, a marker for quiescent and activated HSCs) and subsequently the DyLight594‐conjugated secondary antibody and DyLight488‐conjugated secondary antibody. The nuclei were counterstained with Hoechst 33342 (blue fluorescence). The representative images were captured with the fluorescence microscope, scale bar 25 μm. Arrowheads indicated examples of positively stained cells. The total HSCs (SYP‐positive HSCs, green fluorescence) and 4‐HNE‐, p‐AKT‐, or GATA3‐positive HSCs (red fluorescence) were counted in six randomly chosen fields at 100‐fold magnification and the values were expressed as fold changes relative to the respective total HSCs (empty column). The values were shown as a histogram on the right panel. *P < 0.05.

For investigating the effects of leptin on the levels of 4‐HNE, p‐AKT and GATA3 in HSCs *in vivo*, ob/ob mouse model of TAA‐induced liver injury was used as described in materials and methods. Mice were treated with TAA plus vehicle or leptin for 4‐week, double fluorescence staining was performed with liver sections by using the respective antibody. Figure 5 showed that leptin treatment increases the number of 4‐HNE‐, p‐AKT‐ and GATA3‐positive HSCs, suggesting that leptin up‐regulated the levels of 4‐HNE, p‐AKT, and GATA3 in HSCs in the model. Since NADPH oxidase has emerged as a primary source of reactive oxygen species (ROS) 28 and activated HSCs express the NADPH oxidase as the key enzyme for ROS production 24, 29, the promotion effect of leptin on the level of 4‐HNE (a general marker of ROS) implied a positive role of leptin in NADPH oxidase activity.

As we have demonstrated that leptin stimulated β‐catenin and Shh pathways and reduced PPARγ level in HSCs in the same model 15, we have not detected them again.

Inhibition of leptin‐induced NADPH oxidase pathway reduces the levels of 4‐HNE, p‐AKT, Shh, β‐catenin, and GATA3, companied with the increase in PPARγ levels, in HSCs in ob/ob mouse model of TAA‐induced liver injury.

Since NADPH oxidase pathway mediated leptin‐induced Shh pathway (Fig. 4C), β‐catenin pathway, and PI3K/AKT pathway *in vitro* 26, we next examined whether blockade of NADPH oxidase pathway affected the three pathways in HSCs in the mouse model, and detected the levels of GATA3 and PPARγ in HSCs in the same model. The mice were treated with DPI or vehicle throughout the 4‐week period of treatment with TAA plus leptin. The positive HSCs for 4‐HNE, Shh, p‐AKT, β‐catenin, GATA3, and PPARγ in HSCs in the livers were detected by double fluorescence staining. Results demonstrated that NADPH oxidase inhibitor markedly attenuated the number of the positive HSCs for 4‐HNE, Shh, p‐AKT, β‐catenin, or GATA3, and increased the number of PPARγ‐positive HSCs as compared with the respective control with vehicle (The positive HSCs for β‐catenin and PPARγ were showed in Data S2). These results suggested that, *in vivo*, NADPH oxidase pathway was also required for leptin induction of Shh pathway, PI3K/AKT pathway, β‐catenin pathway and GATA3, companied with decrease in PPARγ levels, in HSCs in the mouse model. Sirius red staining indicated that inhibition of leptin‐induced NADPH oxidase pathway reduced the collagen level in the same model (Data S2).

## Discussion

The research demonstrates novel molecular evens underlying leptin inhibition of PPARγ1 expression in HSCs. Leptin can induce GATA3 expression in HSCs *in vivo* and *in vitro*. GATA3 binds to GATA2‐binding site (around position −2323) on PPARγ1 promoter and interacts with GATA2, contributing to leptin inhibition of PPARγ1 expression in HSCs. Leptin regulates GATA3 expression, at least, through NADPH oxidase pathway, β‐catenin pathway, PI3K/AKT pathway and Shh pathway. There exist a crosstalk between NADPH oxidase pathway and the other pathways.

Our previous research has indicated that GATA2 mediated leptin inhibition of PPARγ1 expression by binding to PPARγ1 promoter at a site around −2323 in HSCs 15. GATA3 was showed to bind to the same site and inhibited PPARγ1 expression. Therefore, leptin could regulate PPARγ1 expression, at least, through both GATA3 and GATA2 in HSCs, which revealed a novel mechanism underlying leptin inhibition of PPARγ1 expression in HSCs. Interestingly, Tong and colleague demonstrated that GATA3 was also able to bind to GATA2‐binding site on PPARγ2 and inhibited adipocyte differentiation 17, but the sequence of GATA2‐binding site in PPARγ1 promoter (Data S1) is different from that in PPARγ2 promoter shown by Tong 17. Our results revealed that GATA3 could interact with GATA2 by binding GATA2. This interaction between GATA3 and GATA2 might contribute to the fine regulation of PPARγ1 expression in HSCs.

Several of signalling pathways were involved in leptin up‐regulation of GATA3 in HSCs *in vitro*, including NADPH oxidase pathway, β‐catenin pathway, PI3K/AKT pathway, and Shh pathway. The *in vivo* experiments demonstrated that leptin induced NADPH oxidase and PI3K/AKT pathways, which were in parallel with leptin‐induced increase in GATA3 expression in HSCs in ob/ob mouse model of TAA‐induced liver injury (Fig. 5). Moreover, interruption of leptin‐induced NADPH oxidase pathway reduced PI3K/AKT pathway, Shh pathway, and β‐catenin pathway, companied by the decrease in GATA3 expression in HSCs in the model. Thereby, these *in vivo* and *in vitro* results suggested that leptin increased GATA3 expression, at least, through the four signalling pathways in HSCs.

NADPH oxidase pathway appeared to be associated with the other three signalling pathways. We showed that leptin‐induced NADPH oxidase pathway could activate Shh pathway, PI3K/AKT pathway, and β‐catenin pathway in HSCs. These results enhanced the notion that NADPH oxidase might be a crucial mediator of fibrogenic actions of leptin 26, implying that NADPH oxidase pathway could be considered as a possible target for inhibiting leptin‐induced liver fibrosis.

The effect of Shh on GATA3 might depend on the cell type, because Shh signalling can negatively regulate GATA3 expression in developing thymocytes 30. The results that the activation of β‐catenin signalling increases GATA3 expression and inhibits adipogenesis in preadipocytes 31 are consistent with the role of β‐catenin signalling in GATA3 expression in HSCs (Fig. 4). Our previous data indicated that PI3K/AKT pathway contributed to the inhibitory effect of leptin on PPARγ expression in HSCs 22 whereas the present investigation revealed the correlation of PI3K/AKT pathway with leptin inhibition of PPARγ1 (a major subtype of PPARγ in HSCs) in HSCs, thus uncovering a detailed target for leptin‐induced PI3K/AKT pathway in HSCs.

**Figure 6:**
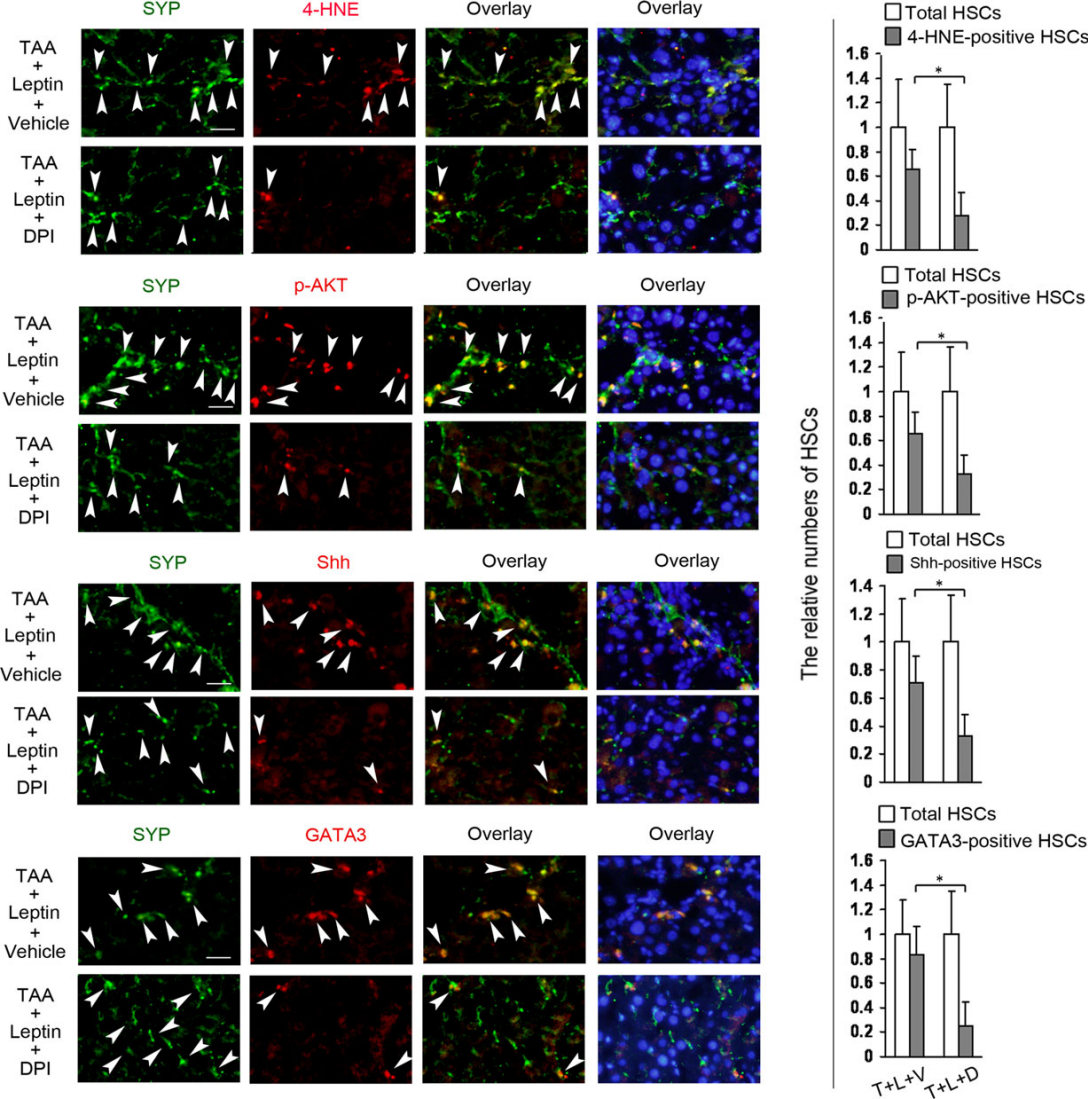
Inhibition of leptin‐induced NADPH oxidase pathway reduces the levels of 4‐HNE, p‐AKT, Shh, and GATA3 in HSCs in ob/ob mouse model of TAA‐induced liver injury. Two groups of ob/ob mice (6 mice/each group) were received DPI (1 μg/g body weight, once per day) or vehicle by i.p. throughout the 4‐week period of treatment with TAA plus leptin (TAA+Leptin+Vehicle = T+L+V; TAA+Leptin+DPI = T+L+D). Double fluorescence staining on the section of liver was performed for detecting 4‐HNE‐, p‐AKT‐, Shh‐, or GATA3‐positive HSCs by using the respective primary antibody plus primary antibody against SYP and subsequently the DyLight594‐conjugated secondary antibody and DyLight488‐conjugated secondary antibody. The nuclei were counterstained with Hoechst 33342 (blue fluorescence). The representative images were captured with the fluorescence microscope. Scale bar 25 μm. Arrowheads indicated examples of positively stained cells. The total HSCs (SYP‐positive HSCs, green fluorescence) and 4‐HNE‐, p‐AKT‐, Shh‐, or GATA3‐positive HSCs (red fluorescence) were counted in six randomly chosen fields at 100‐fold magnification and the values were expressed as fold changes relative to the respective total HSCs (empty column). The values were shown as a histogram on the right panel. *P < 0.05.

GATA3 promotes pulmonary fibrosis 16, 32. Dong and colleague indicated that GATA3 contributed to the activation of a T helper 2 cell‐specific signalling pathway which was involved in carbon nanotubes‐induced pulmonary fibrosis 32. In addition, transforming growth factor‐β (TGF‐β), a key factor in organ fibrosis, increases interleukin‐13 synthesis by GATA3 in T‐lymphocytes from patients with systemic sclerosis 33 and GATA3 could physically and functional interact with Smad3, a component of TGF‐β signalling pathway 34. These results seemed to suggest that GATA3 might be also involved in other organ fibrosis. In fact, the above‐mentioned signalling pathways that mediated leptin up‐regulation of GATA3 in HSCs were associated with leptin‐induced liver fibrosis 9, 15, 22, 26. The present researches revealed the inhibitory effect of leptin‐induced GATA3 on PPARγ1 expression in HSCs. In view that PPARγ plays a crucial role in inhibiting HSC activation 11, 12, the inhibitory effect of GATA3 on PPARγ1 suggested that GATA3 might be involved in HSC activation. Our further experiments indicated that knockdown of GATA3 reduced the expressions of α1(I)collagen and α‐SMA (the well‐established marker of HSC activation) in cultured HSCs and showed that the decrease in leptin‐induced GATA3 (Fig. 6) was in parallel with the reduction in collagen level in liver (Data S2). Moreover, leptin treatment led to a significant increase in GATA3 expression in HSCs (Fig. 5) and caused liver fibrogenesis 15 in ob/ob mouse model of TAA‐induced liver injury. These results implied that GATA3 might also contribute to leptin‐induced liver fibrosis.

In conclusion, the researches revealed that leptin regulation of PPARγ1 was also required GATA3 which was mediated by multiple signalling pathways. Leptin‐induced GATA3 bound to GATA2‐ binding site on PPARγ1 promoter and interacted with GATA2. These data demonstrated novel molecular events for leptin inhibition of PPARγ1 expression in HSCs and thus might have potential implications for clarifying the detailed mechanisms underlying liver fibrosis in diseases in which circulating leptin levels are elevated such as in non‐alcoholic steatohepatitis in obese patients.

## Conflict of interest

None declared.

## Supporting information


**Data S1** The primers for PCR, the DNA sequence for EMSA, and the PPARgamma1 promoter.

**Data S2** Inhibition of leptin‐induced NADPH oxidase pathway reduces the β‐catenin and collagen levels and increases PPARγ levels in HSCs in ob/ob mouse model of TAA‐induced liver injury.
