# Supplementary material for: GATA binding protein 3 is correlated with leptin regulation of PPARγ1 in hepatic stellate cells
Source: J Cell Mol Med. 2016 Oct 6;21(3):568–78. doi: 10.1111/jcmm.13002 (PMC5323826; doi:10.1111/jcmm.13002)
Supplement: Supplementary file 1 — Data S1 The primers for PCR, the DNA sequence for EMSA, and the PPARgamma1 promoter. [file JCMM-21-568-s001.doc]

**Supplemental data 1**

1. The primers used in real-time PCR were as follows:

Mouse GATA-3:

Forward：5′-CCCTTATCAAGCCCAAGC-3′;

Reverse：5′-CCCATTAGCGTTCCTCCT-3′.

Mouse PPARγ1:

Forward: 5′-TTCTGACAGGACTGTGTGACAGAC-3′;

Reverse: 5′-TCCACAGAGCTGATTCCGAAGTT-3′.

Mouse α-SMA:

Forward: 5′-TCTATGCTAACAACGTCCTG-3′;

Reverse: 5′-TCATGGTGCTGGGTGC-3′.

Mouse α1(I) collagen:

Forward: 5′-CCTGGACAGCCTGGACT-3′;

Reverse: 5′-GGACATCTGGGAAGCAA-3′.

Mouse cyclophilin:

Forward: 5′-ACCAATGGCTCACAGTTC-3′;

Reverse: 5′-ACCTTCCGTACCACATCC-3′.

2. The name and primers for construction of mouse GATA-3 promoter luciferase reporter plasmid (The DNA fragments in blue show the enzyme sites):

PCR length: (-2531~+194);

Forward: 5′-CGACGCGTCGCCCAATTACCCACTGTCA-3′;

Reverse: 5′-CCGCTCGAGCGGGAGTAGCAAGGAGCGTAGAG-3′.

3. The nucleotide sequence of the sense strand for EMSA:

5′-tgccctcttttGATAtgtgcagactcaacc-3′.

4. The primers for ChIP assay by using PCR:

Forward: 5′-ATTGCCCATAACACCAGT-3′;

Reverse: 5′-TACCAGATGTCTTTCTCAAT-3′.

5. The possible GATA-3 binding sites between -2333 and -2245 of mouse PPARγ1 promoter:

-2323

taccagagaataaatgccctcttttGATAtgtgcagactcaaccacacatgtggtgcacataaatacacaaacaataaaatataacgtg

-2333 GATA2 (GATA3)

aagagcaattgagaaagacatctggtattaacctctggtctctacatgtacctacacatacacatgtgcac······cct······gacgcacagcacc

-2245 +1 +154
