# Supplementary material for: GATA binding protein 3 is correlated with leptin regulation of PPARγ1 in hepatic stellate cells
Source: J Cell Mol Med. 2016 Oct 6;21(3):568–78. doi: 10.1111/jcmm.13002 (PMC5323826; doi:10.1111/jcmm.13002)
Supplement: Supplementary file 2 — Data S2 Inhibition of leptin‐induced NADPH oxidase pathway reduces the β‐catenin and collagen levels and increases PPARγ levels in HSCs in ob/ob mouse model of TAA‐induced liver injury. [file JCMM-21-568-s002.doc]

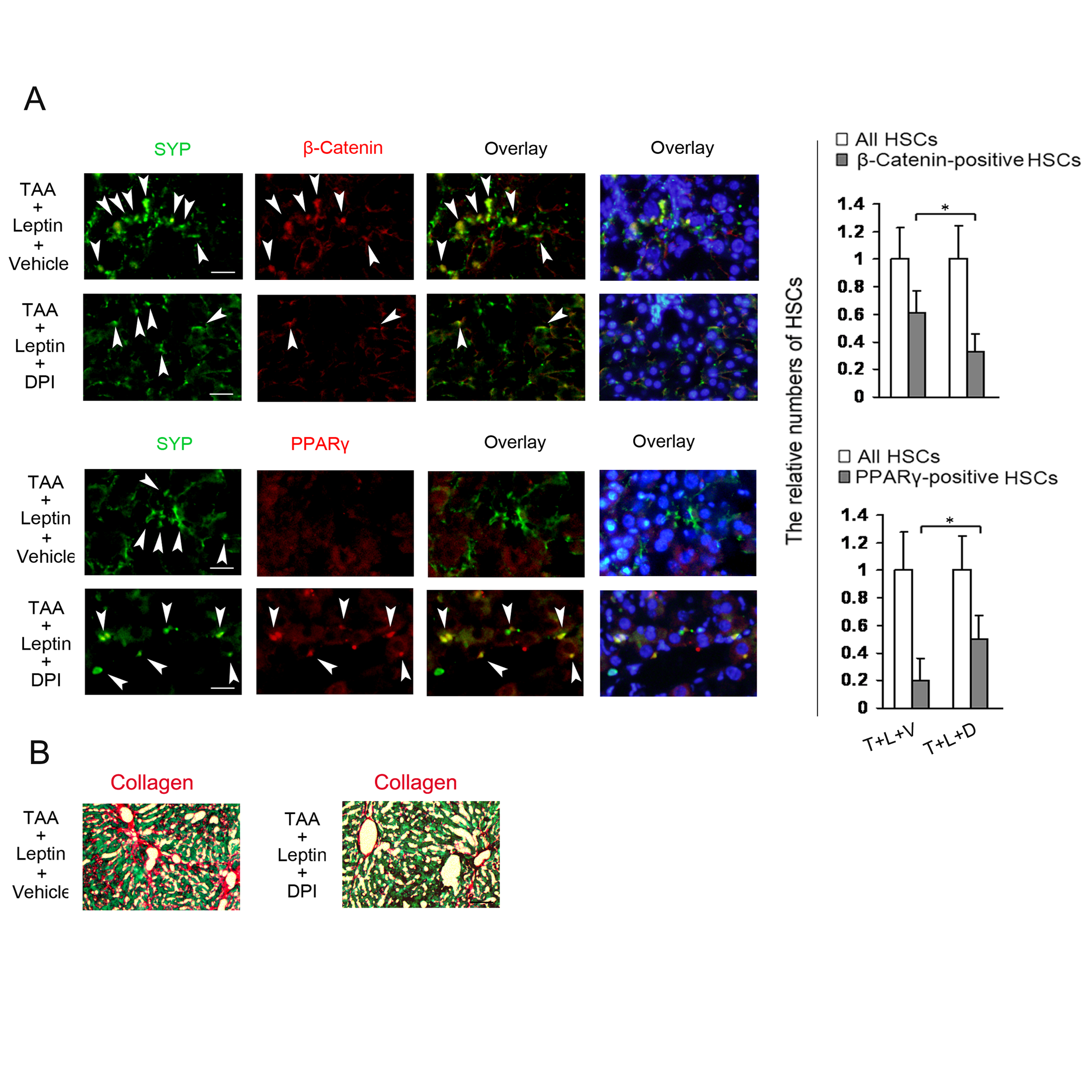


**Suppl. data 2** Inhibition of leptin-induced NADPH oxidase pathway reduces the β-catenin and collagen levels and increases PPARγ levels in HSCs in ob/ob mouse model of TAA-induced liver injury. (**A**) Double fluorescence staining. Two groups of ob/ob mice (6 mice/each group) were treated as shown in Fig. 6. Double fluorescence staining on the section of liver was performed for detecting β-catenin- or PPARγ-positive HSCs by using the respective primary antibody plus primary antibody against SYP and subsequently the DyLight594-conjugated secondary antibody and DyLight488-conjugated secondary antibody. The nuclei were counterstained with Hoechst 33342 (blue fluorescence). The representative images were captured with the fluorescence microscope. *Scale bar* 25 μm. Arrowheads indicated examples of positively stained cells. The total HSCs (SYP-positive HSCs, green fluorescence) and β-catenin-, or PPARγ-positive HSCs (red fluorescence) were counted in six randomly chosen fields at 100-fold magnification and the values were expressed as fold changes relative to the respective total HSCs (empty column). The values were shown as a histogram on the right panel. ^*^*P* < 0.05. (**B**) Sirius red staining. Sirius red staining was used for staining collagen on the above-mentioned liver sections. Liver sections were stained with picric acid-fast green and then incubated with picric acid–sirius red for 1 h. The images were captured with light microscope and representative images were shown.
